# The Risk of Cancer in Patients with Congenital Heart Disease: A Nationwide Population-Based Cohort Study in Taiwan

**DOI:** 10.1371/journal.pone.0116844

**Published:** 2015-02-23

**Authors:** Yu-Sheng Lee, Yung-Tai Chen, Mei-Jy Jeng, Pei-Chen Tsao, Hsiu-Ju Yen, Pi-Chang Lee, Szu-Yuan Li, Chia-Jen Liu, Tzeng-Ji Chen, Pesus Chou, Wen-Jue Soong

**Affiliations:** 1 Division of General Pediatrics, Department of Pediatrics, Taipei Veterans General Hospital, Taipei, Taiwan; 2 Department of Pediatrics, National Yang-Ming University School of Medicine, Taipei, Taiwan; 3 Institute of Public Health and Community Medicine Research Center, National Yang-Ming University School of Medicine, Taipei, Taiwan; 4 Division of Nephrology, Department of Medicine, Taipei Veterans General Hospital, Taipei, Taiwan; 5 Department of Medicine, Taipei City Hospital Heping Fuyou Branch, Taipei, Taiwan; 6 Institute of Emergency and Critical Care Medicine, National Yang-Ming University School of Medicine, Taipei, Taiwan; 7 Division of Pediatric Hematology and Oncology, Department of Pediatrics, Taipei Veterans General Hospital, Taipei, Taiwan; 8 Division of Pediatric Cardiology, Department of Pediatrics, Taipei Veterans General Hospital, Taipei, Taiwan; 9 Institute of Clinical Medicine, National Yang-Ming University School of Medicine, Taipei, Taiwan; 10 Division of Hematology and Oncology, Department of Medicine, Taipei Veterans General Hospital, Taipei, Taiwan; 11 Department of Family Medicine, Taipei Veterans General Hospital, Taipei, Taiwan; MD Anderson Cancer Center, UNITED STATES

## Abstract

**Background:**

The relationship between congenital heart disease (CHD) and malignancies has not been determined. This study aimed to explore the association of CHD with malignancies and examine the risk factors for the development of cancer after a diagnosis of CHD.

**Patients and Methods:**

This nationwide, population-based cohort study on cancer risk evaluated 31,961 patients with newly diagnosed CHD using the Taiwan National Health Insurance Research Database (NHIRD) between 1998 and 2006. The standardized incidence ratios (SIRs) for all and specific cancer types were analyzed, while the Cox proportional hazard model was used to evaluate risk factors of cancer occurrence.

**Results:**

Among patients with newly diagnosed CHD regardless of ages, 187 (0.6%) subsequently developed cancers after a diagnosis of CHD. Patients with CHD had increased risk of cancer (SIR, 1.45; 95% CI, 1.25–1.67), as well as significantly elevated risks of hematologic (SIR, 4.04; 95% CI, 2.76–5.70), central nervous system (CNS) (SIR, 3.51; 95% CI, 1.92–5.89), and head and neck (SIR, 1.81; 95% CI, 1.03–2.94) malignancies. Age (HR, 1.06; 95% CI, 1.05–1.06) and co-morbid chronic liver disease (HR, 1.91; 95% CI, 1.27–2.87) were independent risk factors for cancer occurrence among CHD patients.

**Conclusion:**

Patients with CHD have significantly increased cancer risk, particularly hematologic, CNS, and head and neck malignancies. Physicians who care for patients with CHD should be aware of their predisposition to malignancy after the diagnosis of CHD. Further studies are warranted to clarify the association between CHD and malignancies.

## INTRODUCTION

Congenital heart disease (CHD) is a gross structural abnormality of the heart or intra-thoracic great vessels that is present at birth and manifests as with actual or potential functional significance [1]. It is one of the most common major congenital anomalies, with a reported birth prevalence that varies widely worldwide of 5 to 8 per 1,000 live births [2–7]. Aside from cardiac complications like heart failure, arrhythmia, infectious endocarditis, pulmonary arterial hypertension, and sudden cardiac death [8–10], non-cardiac co-morbidities may also influence the health of patients with CHD [11,12]. Congenital heart disease is a major global health problem [2] and many of the affected patients require specialist follow-up even into adulthood [8].

Congenital anomaly and cancer may have some shared genetic and/or environmental factors that may influence the risk of occurrence. A malformation may also cause physiologic or lifestyle changes that may impact on cancer risk [13,14]. Dysregulation of human development probably plays a vital role in the etiology of cancer among patients with birth defects [15–17]. Previous studies have shown that patients with congenital anomalies have increased risk of developing cancer, such as leukemia, lymphoma, brain tumor, neuroblastoma, germ cell tumor, retinoblastoma, and soft tissue sarcoma [14–16,18–25]. Most of these studies have focused on the association of all categories of congenital anomalies with cancer. Congenital cardiovascular anomaly, a major subgroup of congenital anomalies, is the most frequent type of birth defects that also have a cancer diagnosis [20]. A few studies discuss the individual association of CHD with cancer occurrence, but not all categories of congenital anomalies are included and the results have been conflicting [14,20,26–29]. Furthermore, previous studies do not explore the association between age at CHD diagnosis, gender, duration of follow-up, co-morbidities, and medical radiation examination among CHD patients, and cancer risk.

The National Health Insurance Research Database (NHIRD) in Taiwan offers a nationwide population-based database for research purposes. All of the patients with a diagnosis of CHD or malignancy register with the NHIRD and obtain a catastrophic illness certification, which helps reduce their medical expenditures under the National Health Insurance (NHI) program. These features make the NHIRD appropriate for analyzing the risk of developing cancer [30–40]. To date, there has been no large-scale study examining various malignancies reported in patients with CHD. The aim of this study was to use the nationwide population-based database to explore the association between CHD and malignancies, including all and specific cancer types, and to examine the risk factors for cancer after a diagnosis of CHD.

## MATERIALS AND METHODS

### Ethics statement

The Institutional Review Board of Taipei Veterans General Hospital, Taiwan approved the study. Because all personal identifying information had been encrypted before the database was released, the review board requirement for written informed consent was waived.

### Data sources

This study was based on data from the NHIRD released by the National Health Research Institute (NHRI). Taiwan began the NHI program in 1995, to provide comprehensive health care for all its inhabitants. By the end of 2012, the total population of Taiwan was around 23.3 million. Enrollment in the NHI program is mandatory and there are presently more than 23 million enrollees, representing approximately 99% of Taiwan’s population [36]. The NHI program offers integrated medical care, including out-patient, in-patient, emergency, dental, and traditional Chinese medicine services, as well as drug prescriptions. The NHIRD includes the entire registry and claims data from the NHI system, ranging from demographic data to detailed orders from ambulatory and inpatient care. The NHIRD is managed and publicly released by the NHRI, and contains registration files and original reimbursement claims data for all enrollees in Taiwan. These features make the NHIRD one of the largest and most complete nationwide health care service datasets in the world. The diagnostic codes of the patients in the NHIRD are in the format of the International Classification of Diseases, Ninth Revision, Clinical Modification (ICD-9-CM), and are established by board-certified physicians in their corresponding specialties. The diagnostic accuracy for the major diseases in the NHIRD has been well validated [41,42].

In order to avoid severe financial load on families coping with major illness, the NHI specified 30 categories of catastrophic illnesses (e.g., CHD, cancer, chronic renal failure, autoimmune diseases, and congenital anomalies). Patients with catastrophic illnesses were free from co-payments under the NHI program. Thus, if a patient was diagnosed with one category of the catastrophic illnesses, the attending physician submitted related information in application for a catastrophic illness certificate. The catastrophic illness certificate could not be identified without authorization.

Information about the enrollment and medical utilization for all patients with catastrophic illnesses were included in the NHIRD. All information that might potentially identify any individual patient was encrypted before the database was released. The confidentiality of the database was in accordance with the data regulations of the Bureau of NHI and the NHRI, Taiwan. The NHRI guarded the privacy of all beneficiaries and provided health insurance data to researchers who obtained ethical approval.

### Patient selection

A retrospective cohort study was conducted from January 1, 1998 to December 31, 2006. As in earlier epidemiologic studies of CHD, the ICD-9-CM diagnostic codes used for CHD in the study did not include isolated arrhythmia, cardiomyopathy, Marfan’s syndrome, mitral valve prolapse, and cardiac tumor [4,12] which were secondary to other etiology or minor structural anomaly without functional significance. Using the CHD diagnostic codes (ICD-9-CM codes 745.X, 746.X, 747.0-4) (see S1 Appendix) in the Catastrophic Illness Patient Database, 48758 patients with CHD were identified. Patients with previous diagnosis of CHD (n = 14271), with malignancy upon initial diagnosis of CHD (n = 92), who developed malignancy within 90 days [43], or with a shorter than 90-day follow-up period (n = 2434) were excluded. The final CHD study cohort consisted of 31,961 patients with no previous history of malignancy.

The registration date for CHD in the Catastrophic Illness Patient Database was defined as the index date. Information regarding the age at CHD diagnosis, gender, co-morbidities, and medical radiation exposure was collected for analysis. The types of co-morbidity among CHD patients were as described in previous studies [11,12], defined as those diagnosed before the CHD diagnosis, and identified from the same database by using the ICD-9-CM diagnostic codes [44,45] (see S1 Appendix). All of the enrolled study subjects were followed-up until the diagnosis of cancer, death, or December 31, 2007, whichever was earlier.

### Cancer risk analysis

The diagnosis of cancer in the CHD study cohort was identified using the records of the same Catastrophic Illness Patient Database. The diagnostic codes of cancers were defined as those from 140 to 208.91 in the ICD-9-CM format (see S1 Appendix). Malignant neoplasm of ill-defined sites (ICD-9-CM 195) and secondary cancers (ICD-9-CM 196–199) were excluded because the aim of the current study was to investigate the risk of primary cancers. The main dependent variable in this study was cancer incidence. The registration time for cancer in the Catastrophic Illness Patient Database was chosen for incidence analysis. Stratified analyses of standardized incidence ratios (SIRs) according to gender, age at the time of CHD diagnosis, and follow-up period after the CHD diagnosis, were conducted to estimate the risk of primary cancer in patients with CHD.

### Statistical analysis

The person-years for cancer risk were recorded from the index date to the date of cancer diagnosis, death, or the end of 2007, whichever was earlier. The incidence densities (per 100,000 person-years) of cancer occurrence in the CHD cohort were then calculated. The association between CHD and cancers by SIRs was examined. The SIRs were calculated as the number of observed cancer cases among the CHD cohort divided by the number of expected cancer cases according to the national age-, gender-, and period-specific cancer rates from the yearly reports of cancer rates from the Taiwan Cancer Registry. The registry provided a database of cancer-related data for various research efforts and was made available upon request (S1 Table). The 95% confidence interval (CI) of SIR was calculated using Byar’s approximation [46].

The Cox proportional hazard model with hazard ratio (HR) was used to analyze the risk factors for the occurrence of cancer. Control variables such as age, gender, co-morbidities, and medical radiation examination were included in the model.

Microsoft Office Excel 2003 (Microsoft Corporation, Redmond, Washington, USA) and the SPSS statistical software version 19.0 for windows (SPSS Inc., Chicago, IL, USA) were used to perform the statistical analysis. Statistical significance was set at *p*<0.05.

## RESULTS

### Demographic and clinical characteristics

**Fig 1:**
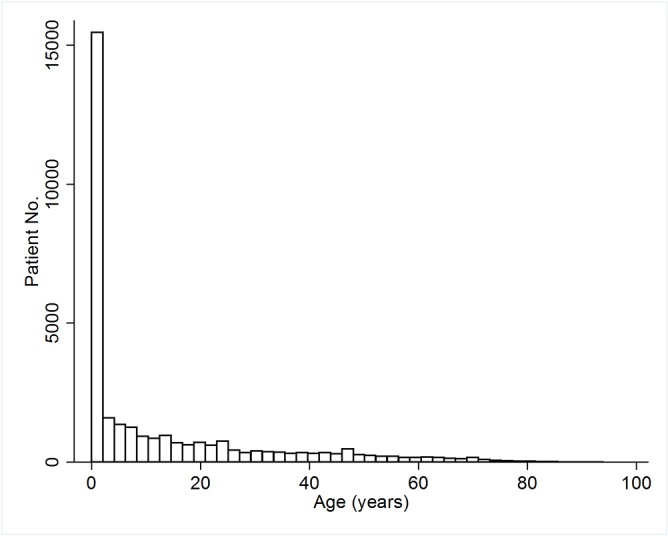
Distribution of age at diagnosis of congenital heart disease (CHD).

During the study period, 31,961 of the patients were enrolled, including 15,156 (47.4%) males and 16,805 (52.6%) females, for a male to female gender ratio of 0.9. Their median age upon CHD diagnosis was 3.5 years and the age distribution at CHD diagnosis is shown in Fig. 1. Heart failure (n = 4149; 13.0%) was the most common co-morbidity. There were 15,613 (48.9%) patients who underwent cardiac catheterizations and 6,051 (18.9%) who had computed tomography (CT) examinations while the CHD diagnosis was being established. The median follow-up period in this study was 5.3 years. The detailed demographic and clinical characteristics about the study population are shown in Table 1.

**Table 1:** Demographic and clinical characteristics of patients with CHD but without any previous history of malignancy.

	Total	Male	Female	*p*
No. of patients, n (%)	31,961	15,156 (47.4)	16,805 (52.6)	
Age at CHD diagnosis, years, n (%)				
0–5	17,678 (55.3)	8,635 (57.0)	9,043 (53.8)	
6–17	5,676 (17.8)	2,754 (18.2)	2,922 (17.4)	
18–44	5,722 (17.9)	2,542 (16.8)	3,180 (18.9)	
45–64	2,184 (6.8)	929 (6.1)	1,255 (7.5)	
≥ 65	701 (2.2)	296 (2.0)	405 (2.4)	
Median age at diagnosis, years (IQR)	3.5 (0.2–19.7)	2.9 (0.2–17.8)	4.1 (0.3–22.0)	<0.001
Median follow-up period, years (IQR)	5.2 (2.7–7.6)	5.3 (2.7–7.6)	5.2 (2.7–7.5)	0.422
Person-years at cancer risk	163,430	77,696	85,734	
Co-morbidity, n (%)				
Heart failure	4,149 (13.0)	1,929 (12.7)	2,220 (13.2)	0.200
Chronic pulmonary disease	3,864 (12.1)	1,774 (11.7)	2,090 (12.4)	0.045
Scoliosis	2,009 (6.3)	1,063 (7.0)	946 (5.6)	<0.001
Cerebrovascular disease	1,446 (4.5)	745 (4.9)	701 (4.2)	0.001
Chronic liver disease	1,182 (3.7)	570 (3.8)	612 (3.6)	0.573
Diabetes mellitus	805 (2.5)	329 (2.2)	476 (2.8)	<0.001
Chromosomal anomaly	734 (2.3)	346 (2.3)	388 (2.3)	0.877
Epilepsy	598 (1.9)	321 (2.1)	277 (1.6)	0.002
Peripheral vascular disease	411 (1.3)	237 (1.6)	174 (1.0)	<0.001
Chronic kidney disease	409 (1.3)	189 (1.2)	220 (1.3)	0.622
Congenital respiratory anomaly	353 (1.1)	193 (1.3)	160 (1.0)	0.006
Mental retardation	304 (1.0)	159 (1.0)	145 (0.9)	0.087
Rheumatologic disease	223 (0.7)	63 (0.4)	160 (1.0)	<0.001
Cerebral palsy	224 (0.7)	111 (0.7)	113 (0.7)	0.521
Catheterization	15,613 (48.9)	7,242 (47.8)	8,371 (49.8)	<0.001
CT scan	6,051 (18.9)	3,113 (20.5)	2,938 (17.5)	<0.001

Abbreviations: CHD, Congenital heart disease; CT, computed tomography; IQR, inter-quartile range

*p* value, statistically significant difference between male and female patients with CHD

### Cancer risk in CHD patients without a previous history of malignancy

Among the 31,961 patients with CHD of all ages, 187 (0.6%) were diagnosed with cancer, with the median time to a cancer diagnosis of 3.0 years. The person-years for cancer risk were 163,430 and the incidence density for patients for malignancy after a CHD diagnosis was 114.4 cases per 100,000 person-years, with 105.5 and 122.5 for male and female CHD patients, respectively.

The SIRs for cancers after a CHD diagnosis are detailed in Table 2. There was a significantly increased risk of cancer among patients with CHD (SIR, 1.45; 95% CI, 1.25–1.67). When stratified by gender, the significantly increased all cancer risk remained in both males (SIR, 1.46; 95% CI, 1.16–1.81) and females (SIR, 1.44; 95% CI, 1.17–1.74). Stratifying patients by age at the time of CHD diagnosis, cancer risk was highest in those aged 0–5 years (SIR, 1.79; 95% CI, 1.22–2.46). During the follow-up period, cancer occurrence was significantly increased at 1–2 years (SIR, 1.51; 95% CI, 1.18–1.91) and ≥3 years (SIR, 1.63; 95% CI, 1.32–2.01) after the diagnosis of CHD. The strength of this association did not change with the inclusion and exclusion of data from the first year of follow-up and remained statistically significant (Table 3).

**Table 2:** Standardized incidence ratios (SIR) for all cancers according to gender, age at CHD diagnosis, and follow-up period.

	Total		Male		Female	
Characteristics	O/E	SIR (95% CI)	O/E	SIR (95% CI)	O/E	SIR (95% CI)
All cancers	187/129.29	1.45 (1.25–1.67)	82/56.13	1.46 (1.16–1.81)	105/73.16	1.44 (1.17–1.74)
Age at CHD diagnosis, years						
0–5	34/19.33	1.79 (1.22–2.46)	14/10.28	1.36 (0.74–2.28)	20/9.05	2.21 (1.35–3.41)
6–17	5/3.23	1.55 (0.50–3.62)	1/1.71	0.59 (0.01–3.26)	4/1.52	2.64 (0.71–6.75)
18–44	47/26.39	1.78 (1.31–2.37)	16/7.71	2.08 (1.19–3.37)	31/18.68	1.66 (1.13–2.36)
45–64	76/50.03	1.52 (1.20–1.90)	36/21.07	1.71 (1.20–2.37)	40/28.96	1.38 (0.99–1.88)
≥ 65	25/30.31	0.82 (0.53–1.22)	15/15.36	0.98 (0.55–1.61)	10/14.95	0.67 (0.32–1.23)
Follow-up period, years						
< 1	26/28.68	0.85 (0.55–1.24)	9/12.89	0.65 (0.30–1.23)	17/15.79	1.01 (0.59–1.62)
1–2	70/44.92	1.51 (1.18–1.91)	34/19.78	1.66 (1.15–2.33)	36/25.17	1.39 (0.98–1.93)
≥ 3	91/55.69	1.63 (1.32–2.01)	39/23.47	1.66 (1.18–2.27)	52/32.21	1.61 (1.21–2.12)

Abbreviations: CI, confidence interval; E, expected case number; O, observed case number; SIR, standardized incidence ratio

**Table 3:** Sensitivity analysis of the standardized incidence ratios (SIR) with 95% CI for the association between CHD and all cancer risk, with the inclusion and exclusion of data from first year of follow-up.

	Total		Male		Female	
Follow-up period, years	O/E	SIR (95% CI)	O/E	SIR (95% CI)	O/E	SIR (95% CI)
Include data from the first year	187/129.29	1.45 (1.25–1.67)	82/56.13	1.46 (1.16–1.81)	105/73.16	1.44 (1.17–1.74)
Exclude data from the first year	161/100.61	1.60 (1.36–1.87)	73/43.24	1.69 (1.32–2.12)	88/57.37	1.53 (1.23–1.89)

Abbreviations: CI, confidence interval; E, expected case number; O, observed case number; SIR, standardized incidence ratio

With regards to specific cancer types, the risk of hematologic malignancies (SIR, 4.04; 95% CI, 2.76–5.70), central nervous system (CNS) tumors (SIR, 3.51; 95% CI, 1.92–5.89), and head and neck tumors (SIR, 1.81; 95% CI, 1.03–2.94) were significantly higher in patients with CHD. There were no gender differences in cancer risk among the CHD patients, but became significant when the risk of developing specific cancer types was calculated. Male CHD patients were more likely to have thyroid (SIR, 6.37; 95% CI, 1.28–18.61), hematologic (SIR, 4.08; 95% CI, 2.28–6.74), and CNS (SIR, 3.44; 95% CI, 1.26–7.49) malignancies, whereas female CHD patients had significantly higher risks of developing hematologic (SIR, 4.00; 95% CI, 2.33–6.41), CNS (SIR, 3.57; 95% CI, 1.54–7.03), and uterine (SIR, 4.16; 95% CI, 1.52–9.06) malignancies. The SIRs for all and specific cancer types among CHD patients who had no previous history of malignancy are listed in Table 4.

**Table 4:** Standardized incidence ratios (SIRs) for all and specific cancer types among patients with CHD.

	Total		Male		Female	
Site of cancers	O/E	SIR (95% CI)	O/E	SIR (95% CI)	O/E	SIR (95% CI)
All cancers	187/129.3	1.45 (1.25–1.67)	82/56.1	1.46 (1.16–1.81)	105/73.2	1.44 (1.17–1.74)
Hematologic malignancies	32/8.0	4.04 (2.76–5.70)	15/3.7	4.08 (2.28–6.74)	17/4.3	4.00 (2.33–6.41)
CNS	14/4.0	3.51 (1.92–5.89)	6/1.7	3.44 (1.26–7.49)	8/2.2	3.57 (1.54–7.03)
Head and neck	16/8.8	1.81 (1.03–2.94)	12/6.8	1.76 (0.91–3.08)	4/2.0	1.97 (0.53–5.06)
Thyroid	4/3.1	1.30 (0.35–3.32)	3/0.5	6.37 (1.28–18.61)	1/2.6	0.38 (0.01–2.13)
Lung and mediastinum	12/10.7	1.12 (0.58–1.96)	6/6.5	0.93 (0.34–2.02)	6/4.3	1.41 (0.52–3.07)
Digestive	50/38.4	1.30 (0.97–1.72)	27/21.2	1.27 (0.84–1.85)	23/17.2	1.34 (0.85–2.00)
Stomach	6/5.3	1.13 (0.41–2.47)	4/2.9	1.36 (0.37–3.48)	2/2.4	0.85 (0.10–3.07)
Colon and rectum	15/12.0	1.25 (0.70–2.07)	10/5.6	1.80 (0.86–3.31)	5/6.4	0.78 (0.25–1.82)
Liver and biliary tract	24/15.9	1.51 (0.97–2.24)	12/9.9	1.22 (0.63–2.12)	12/6.1	1.98 (1.02–3.46)
Breast	19/11.4	(1.00–2.60)	-	-	19/11.4	1.67 (1.00–2.60)
Genitourinary	33/29.1	1.14 (0.78–1.60)	10/7.2	1.39 (0.67–2.56)	23/21.9	1.05 (0.67–1.58)
Cervix	8/15.1	(0.23–1.04)	-	-	13/15.1	0.86 (0.46–1.47)
Uterus	6/1.4	(1.52–9.06)	-	-	6/1.4	4.16 (1.52–9.06)
Prostate	4/2.0	1.98 (0.53–5.06)	4/2.0	(0.53–5.06)	-	-
Bladder	5/2.5	2.03 (0.66–4.75)	4/1.5	2.67 (0.72–6.84)	1/1.0	1.04 (0.01–5.79)
Kidney	5/3.8	1.31 (0.42–3.06)	1/2.0	0.49 (0.01–2.74)	4/1.8	2.25 (0.61–5.77)

Abbreviations: CHD, Congenital heart disease; CNS, central nervous system; CI, confidence interval; E, expected case number; O, observed case number; SIR, standardized incidence ratio.

The cancers with less than 4 observed cases are not showing, including esophagus (n = 1), pancreas (n = 2), ovary (n = 2), bone and soft tissue (n = 2), skin (n = 3), and others (n = 2).

### Risk factors for cancer in patients after the diagnosis of CHD

By Cox univariate proportional hazard analysis, there was increased cancer risk in CHD patients with one of the following characteristics: older age at CHD diagnosis; co-morbidities with heart failure, cerebrovascular disease, peripheral vascular disease, chronic pulmonary disease, diabetes mellitus, chronic kidney disease, chronic liver disease, or epilepsy; and having received medical radiation examinations like cardiac catheterization or CT. On Cox multivariate proportional hazard analysis, age (HR, 1.06; 95% CI, 1.05–1.06) and co-morbidity with chronic liver disease (HR, 1.91; 95% CI, 1.27–2.87) were independent risk factors of cancer in CHD patients (Table 5).

**Table 5:** Hazard ratio (HR) for risk factors of cancer among patients with CHD.

Variables	Univariate analysis			Multivariate analysis^a^		
	HR	(95% CI)	*p*	HR	(95% CI)	*p*
Age (years)	1.06	(1.05–1.06)	<0.001	1.06	(1.05–1.06)	<0.001
Male gender	0.87	(0.65–1.16)	0.349			
Co-morbidity						
Heart failure	2.98	(2.15–4.13)	<0.001	1.13	(0.79–1.61)	0.511
Cerebrovascular disease	7.17	(4.97–10.34)	<0.001	1.32	(0.67–2.59)	0.423
Chronic pulmonary disease	3.37	(2.40–4.73)	<0.001	0.94	(0.65–1.37)	0.754
Diabetes mellitus	10.29	(6.82–15.54)	<0.001	1.26	(0.80–1.98)	0.326
Chronic kidney disease	8.90	(5.05–15.67)	<0.001	1.56	(0.86–2.84)	0.142
Chronic liver disease	8.08	(5.44–12.00)	<0.001	1.91	(1.27–2.87)	0.002
Rheumatologic disease	1.15	(0.16–8.23)	0.888			
Chromosomal anomaly	1.75	(0.77–3.94)	0.179			
Cerebral palsy	1.80	(0.45–7.26)	0.408			
Mental retardation	0.05	(0.00–80.25)	0.452			
Epilepsy	2.69	(1.26–5.72)	0.010	1.48	(0.66–3.32)	0.338
Scoliosis	0.83	(0.43–1.63)	0.592			
Congenital respiratory anomaly	0.05	(0.00–49.67)	0.393			
Catheterization	1.13	(1.04–1.23)	0.004	0.96	(0.88–1.04)	0.313
CT scan	1.16	(1.11–1.20)	<0.001	0.99	(0.92–1.07)	0.861

^a^All of the factors with *p*<0.1 in univariate analyses were included in the Cox multivariate analysis.

Abbreviations: CI, confidence interval; HR, hazard ratio

## DISCUSSION

To date, this is the first large-scale nationwide population-based analysis with the largest sample size to investigate the cancer risk in a population diagnosed with CHD without any previous history of malignancies. The study, which includes 31,961 patients with newly diagnosed CHD of all ages, demonstrates a significantly elevated cancer risk, with an SIR of 1.45 after a median 5.3-year follow-up period. In addition, there is evidence of a significant association between CHD and specific cancer types, including hematologic, CNS, and head and neck malignancies.

The validity of the results here may be strongly reinforced by the study design, which includes precise diagnostic criteria and longitudinal follow-up in time. Furthermore, the certification of Catastrophic Illnesses, including CHD and cancer, can exempt patients from related medical expenditures under Taiwan’s NHI system. As a result, the verification of catastrophic illness is very strict. For CHD, supportive medical records and examination reports like echocardiograms and catheterization are required. For malignancies, histologic or cytologic evidence is required. These features make the diagnoses of CHD and malignancy in this study robust and reliable.

The median age of CHD diagnosis in the study population is 3.5 years, with an inter-quartile range of 0.2 to 19.7 years. This may be because CHD is not always diagnosed in early childhood, as certain CHD conditions are detected only in adulthood, when symptoms are manifested [9,10,47]. Age upon CHD diagnosis is dependent on the severity of the congenital cardiovascular anomaly. Despite the wide range of age at CHD diagnosis, the results of this study still reveal a significantly greater risk of cancer risk in these patients. Physicians who take care of patients with CHD should be aware of their predisposition to malignancy.

The median follow-up duration in these CHD patients until a diagnosis of cancer is 3 years. Most cancer cases in this study cohort have been detected ≥1 year after the diagnosis of CHD. The sensitivity analysis about the strength of this association between CHD and cancer risk remain statistically significant with the inclusion and exclusion of data from the first year of follow-up. Stratifying patients by age at the time of CHD diagnosis, cancer risk is highest in those aged 0–5 years. The risk for cancer occurrence in individuals with other diseases, such as chronic urticaria, rheumatoid arthritis, and primary Sjögren’s syndrome, as reported in previous studies, is the highest within the first year after the diagnosis has been established [36,37,48], which is different from the results of the current study. In this study, the cancer risk within 1 year after CHD diagnosis is not statistically significant (SIR, 0.85; 95% CI, 0.55–1.24).This may be due to the median diagnostic age of CHD in this study (3.5 years) is lower than those for other diseases explored in literature on cancer risk. The time interval that is needed for oncogenic factors to have an effect in younger CHD patients may be the reason for the differences in the duration of follow-up before cancer detection.

Most of the previous studies have focused on the association between all congenital anomalies and childhood cancer [14–16,18–20]. This study has placed emphasis on all and specific cancer risks in CHD patients of all ages. The association between CHD and specific cancer types has not been well documented before. Carozza et al. reported that cardiac and circulatory anomalies were the most frequent type of congenital birth defects with a cancer diagnosis, of which leukemia was the most common [20]. Previous studies also revealed that hematologic malignancies were the most well-known cancer type to be related to CHD [15,19,20,26]. The results of the present study show a significant increase in the risk for all cancers, with an SIR value of 1.45 after CHD diagnosis. The risk of hematologic malignancies with an SIR value of 4.04 is significantly elevated. Patients with congenital anomalies have an increased risk for various solid tumors [14,15,19,20], and the results of the present study demonstrate a significantly higher risk for CNS and head and neck tumors (SIR, 3.51 and 1.81, respectively).

Narod et al. speculated that gene mutations in embryogenesis may be related to birth defects, cancer type, or both [16]. The mechanism that explains the association between CHD and cancer remains elusive and it is not known whether the concurrence of CHD and various tumors is just a coincidence or whether they share common etiologic factors. Dysregulation during early human development may play an important role in cancer occurrence [15]. Changes in cardiovascular structure or function, lifestyle adaptation related to CHD, or environmental exposures, may mediate the risk of cancer development among patients with CHD. Results of the current study indicate that CHD and cancer may occur in the same individual, through some possible communal underlying factors. Further investigations are warranted to elucidate the association between CHD and malignancy, and to unravel the mechanisms that are involved.

In this study, the SIRs for overall cancer occurrences are not meaningfully different between males and females. Although the results illustrate the greater risk of specific cancer types between different genders, the observed gender differences may be due to the small numbers of cancer cases, which may restrict the power to investigate this difference of specific cancer types among CHD patients. Further investigations about the gender differences of specific cancer sites in CHD patients are also needed.

Due to the dissimilar references used in calculating SIRs, the results cannot be directly compared with the SIRs from previous studies. The SIR values for most cancers in this study are modest and the case numbers are small for certain cancer types, which limit further subclass analyses. The interpretation of these SIRs should be made cautiously and additional studies are needed to clarify these relationships.

After controlling for age, gender, co-morbidities, time to cancer diagnosis, and medical radiation examination, the results show that radiation exposure tests are not independent risk factors to explain the development of cancer in CHD patients. The association between CHD patients and radiation exposure during cardiac catheterization and cancer has been investigated before, and the results are inconsistent [26–29]. Retrospective cohort studies conducted by Spengler et al. [27] and by McLaughlin et al. [28] do not demonstrate a significant increase in cancer occurrence among CHD patients who underwent cardiac catheterization. However, Modan et al. have found the SIR for cancer following cardiac catheterization due to CHD in 674 children to be 2.3 (95% CI, 1.2–4.1) [26]. Most previous reports are hospital-based studies with limited numbers of observation and possible selection bias. Small sample sizes, with limited power, and without control for covariates, may be the possible reasons for the controversial results. In addition, the disagreements may also result from the unclear and complex pathogenic mechanism of interaction between CHD and cancer. Although the current study demonstrates that radiation examinations are not the independent risk factors for cancer occurrence among CHD patients, further study with a longer follow-up period and more information about the dosage of irradiation may be necessary to explore this correlation, especially among younger CHD patients.

Chronic liver disease is the only independent risk factor, other than age, that shows a significant relationship with cancer occurrence in patients with CHD. Because some co-morbidities may require radiation exposure tests, it is possible for over-adjustment if all variables, including co-morbidities, catheterization, and CT scan, are simultaneously involved in the model. However, the analysis for HRs in the model, excluding radiation exposure, shows that age and chronic liver disease remain independent risk factors for cancer occurrence among CHD patients. The observed significant association with co-morbidity and cancer may be due to a heightened medical surveillance in patients with CHD or vice versa. Therefore, additional research is required before any conclusions can be drawn.

Since follow-up medical examinations for CHD patients may be arranged regularly after a CHD diagnosis, the strengthened medical scrutiny may increase the likelihood of early cancer detection among CHD patients. Further exploration regarding the extent of the potential detection bias needs to be examined in order to provide more evidence about the association of CHD with malignancies.

This is the first large-scale nationwide population-based cohort study of cancer occurrence and CHD. The major strengths of the current study are the case definitions, its population-based design, and its complete coverage of CHD and cancer cases in the population, wherein the possibility of loss to follow-up is essentially precluded.

Nevertheless, this study has some limitations that are worth considering. First, because this study is epidemiologic in nature, it cannot establish a causative link between CHD and cancer. Second, personal information, including CHD phenotype and severity, environmental exposure, family history, lifestyle, and habits such as smoking and alcohol use, are not documented in the NHIRD. Therefore, an analysis of the possible relationships between these personal characteristics and the cancer risk is not possible. Third, the follow-up duration is relatively short and it is possible that certain oncogenic factors may need a longer time to have an effect. Additional large studies, with a longer follow-up period, involving different age strata, are needed to clarify possible patho-physiologic mechanisms and make definitive conclusions.

In conclusion, cancer risk, in particular the risk for hematologic, CNS, and head and neck malignancies, is significantly elevated in patients after a diagnosis of CHD. Physicians should be aware of the predisposition to malignancy in patients after a CHD diagnosis. Further studies are needed to clarify the association between CHD and the development of cancer.

## Supporting Information

S1 Appendix The ICD-9-CM diagnostic codes used in the study. (DOC)

S1 Table The cancer incidence rate in Taiwan. (DOC)
