# Supplementary material for: The Risk of Cancer in Patients with Congenital Heart Disease: A Nationwide Population-Based Cohort Study in Taiwan
Source: PLoS One. 2015 Feb 23;10(2):e0116844. doi: 10.1371/journal.pone.0116844 (PMC4338195; doi:10.1371/journal.pone.0116844)
Supplement: S1 Appendix — (DOC) [file pone.0116844.s001.doc]

**Appendix**

The ICD-9-CM diagnostic codes for CHD were as follows: 745.0, common truncus; 745.1, transposition of the great vessels; 745.2, Tetralogy of Fallot; 745.3, common ventricle; 745.4, ventricular septal defect; 745.5, ostium secundum type atrial septal defect; 745.6, endocardial cushion defects; 745.7, cor biloculare; 746.0, anomalies of pulmonary valve congenital; 746.1, tricuspid atresia and stenosis, congenital; 746.2, Ebstein's anomaly; 746.3, congenital stenosis of aortic valve; 746.4, congenital insufficiency of the aortic valve; 746.5, congenital mitral stenosis; 746.6, congenital mitral insufficiency; 746.7, hypoplastic left heart syndrome; 746.8, other specified congenital anomalies of heart; 747.0, patent ductus arteriosus; 747.1, co-arctation of the aorta; 747.2, other congenital anomalies of the aorta; 747.3, congenital anomalies of the pulmonary artery; and 747.4, anomalies of the great veins.

The ICD-9-CM diagnostic codes for co-morbidities were as follows: 398, 402, 428, heart failure; 491-493, chronic pulmonary disease; 737, scoliosis; 430-433, 435, cerebrovascular disease; 571, 572.3, 572.8, chronic liver disease ; 250, diabetes mellitus; 758, chromosome anomaly; 345, epilepsy; 440-447, peripheral vascular disease; 403, 404, 585, 586, chronic kidney disease; 748.4-748.6, congenital respiratory anomaly; 317-319, mental retardation; 710, 714, 725, rheumatologic disease; and 343, cerebral palsy.

The ICD-9-CM diagnostic codes for malignancies were as follows: 200-208, hematologic malignancies; 191, 192, central nervous system; 140-149, 160, 161, head and neck; 193, thyroid; 162-164, lung and mediastinum; 150, esophagus; 151, stomach; 153-154, colon and rectum; 155, 156, liver and biliary tract; 157, pancreas ; 174, breast; 180, cervix; 182, uterus; 183, ovary; 185, prostate; 188, bladder; 189, kidney; 170, 171, bone and soft tissue; and 172, 173, skin.
