# Supplementary material for: The Risk of Cancer in Patients with Congenital Heart Disease: A Nationwide Population-Based Cohort Study in Taiwan
Source: PLoS One. 2015 Feb 23;10(2):e0116844. doi: 10.1371/journal.pone.0116844 (PMC4338195; doi:10.1371/journal.pone.0116844)
Supplement: S1 Table — (DOC) [file pone.0116844.s002.doc]

| **Table S1.** The cancer incidence rate in Taiwan. | | | | | | | | | | | | | | | | | | | | | |
| --- | --- | --- | --- | --- | --- | --- | --- | --- | --- | --- | --- | --- | --- | --- | --- | --- | --- | --- | --- | --- | --- |
|  |  |  | Age (years) | | | | | | | | | | | | | | | | | | |
| Category | ICD-9 | Gender | 0 | 1-4 | 5-9 | 10-14 | 15-19 | 20-24 | 25-29 | 30-34 | 35-39 | 40-44 | 45-49 | 50-54 | 55-59 | 60-64 | 65-69 | 70-74 | 75-79 | 80-84 | 85+ |
| Head and neck | 140-149 | Male | 0 | 0 | 0 | 0.06 | 0.09 | 0.24 | 0.70 | 1.72 | 3.89 | 6.20 | 9.12 | 10.65 | 11.86 | 10.22 | 9.60 | 7.44 | 5.68 | 5.37 | 5.19 |
|  |  | Female | 0.07 | 0.02 | 0 | 0.05 | 0.05 | 0.12 | 0.24 | 0.32 | 0.64 | 0.83 | 1.04 | 1.71 | 1.77 | 1.67 | 2.06 | 1.99 | 2.26 | 2.24 | 1.75 |
| Digestive | 150-159 | Male | 0.26 | 0.05 | 0.14 | 0.07 | 0.21 | 0.44 | 1.01 | 2.36 | 4.41 | 7.90 | 12.90 | 21.02 | 35.60 | 48.99 | 62.99 | 68.67 | 78.50 | 90.29 | 90.27 |
|  |  | Female | 0.50 | 0.16 | 0.03 | 0.07 | 0.13 | 0.29 | 0.62 | 1.10 | 2.17 | 3.36 | 5.50 | 8.44 | 17.27 | 23.52 | 33.01 | 43.76 | 55.23 | 60.87 | 52.97 |
| Esophagus | 150 | Male | 0 | 0 | 0 | 0 | 0 | 0 | 0 | 0.06 | 0.25 | 0.76 | 1.28 | 2.00 | 2.98 | 3.57 | 4.02 | 3.77 | 4.27 | 3.48 | 4.19 |
|  |  | Female | 0 | 0 | 0 | 0 | 0 | 0 | 0 | 0 | 0 | 0.04 | 0.01 | 0.09 | 0.15 | 0.20 | 0.31 | 0.23 | 1.05 | 1.07 | 2.07 |
| Stomach | 151 | Male | 0.07 | 0 | 0 | 0.01 | 0.03 | 0.04 | 0.09 | 0.29 | 0.33 | 0.85 | 1.36 | 2.11 | 3.67 | 5.63 | 9.09 | 13.32 | 17.19 | 21.91 | 24.56 |
|  |  | Female | 0 | 0 | 0 | 0.01 | 0 | 0.02 | 0.13 | 0.25 | 0.50 | 0.75 | 1.14 | 1.39 | 1.94 | 3.41 | 3.62 | 7.38 | 9.76 | 10.13 | 10.21 |
| Colon and rectum | 153-154 | Male | 0 | 0 | 0.01 | 0.01 | 0.04 | 0.16 | 0.28 | 0.55 | 0.95 | 1.61 | 2.70 | 4.41 | 8.73 | 14.17 | 20.76 | 22.87 | 26.31 | 30.87 | 32.95 |
|  |  | Female | 0 | 0 | 0 | 0 | 0.04 | 0.16 | 0.31 | 0.55 | 0.94 | 1.83 | 2.69 | 3.50 | 7.59 | 9.12 | 13.79 | 16.10 | 22.65 | 27.51 | 21.38 |
| Biliary tract | 155-156 | Male | 0.13 | 0.03 | 0.07 | 0.04 | 0.08 | 0.20 | 0.55 | 1.34 | 2.76 | 4.32 | 6.97 | 11.51 | 18.54 | 23.15 | 25.68 | 24.09 | 25.34 | 25.92 | 21.97 |
|  |  | Female | 0.21 | 0.07 | 0 | 0.05 | 0.04 | 0.07 | 0.15 | 0.19 | 0.59 | 0.53 | 1.24 | 2.73 | 6.67 | 9.25 | 12.74 | 17.15 | 16.96 | 17.27 | 14.04 |
| Pancreas | 157 | Male | 0 | 0 | 0 | 0 | 0.02 | 0.01 | 0.02 | 0.07 | 0.07 | 0.20 | 0.39 | 0.65 | 1.13 | 1.96 | 2.85 | 3.67 | 4.13 | 6.95 | 5.19 |
|  |  | Female | 0 | 0 | 0 | 0 | 0.01 | 0.01 | 0 | 0.04 | 0.05 | 0.08 | 0.17 | 0.44 | 0.70 | 0.91 | 1.78 | 2.31 | 3.83 | 3.73 | 4.31 |
| Lung and mediastinum | 162-165 | Male | 0.13 | 0.02 | 0 | 0.07 | 0.06 | 0.14 | 0.14 | 0.24 | 0.67 | 1.16 | 1.89 | 4.27 | 7.57 | 13.15 | 24.75 | 33.37 | 40.88 | 41.19 | 40.54 |
|  |  | Female | 0 | 0.03 | 0 | 0.01 | 0.03 | 0.06 | 0.13 | 0.16 | 0.51 | 0.96 | 1.56 | 2.70 | 4.68 | 6.04 | 9.12 | 13.52 | 16.44 | 17.27 | 18.03 |
| Bone and soft tissue | 169-171 | Male | 0.26 | 0.74 | 0.37 | 0.35 | 0.54 | 0.44 | 0.28 | 0.34 | 0.52 | 0.70 | 0.80 | 0.80 | 1.60 | 2.44 | 3.24 | 3.98 | 4.81 | 4.53 | 6.19 |
|  |  | Female | 0.36 | 0.65 | 0.39 | 0.38 | 0.54 | 0.31 | 0.37 | 0.36 | 0.42 | 0.62 | 0.60 | 0.81 | 1.12 | 1.67 | 2.09 | 2.50 | 3.08 | 1.92 | 2.71 |
| Skin | 173 | Male | 0 | 0.02 | 0.01 | 0.07 | 0.03 | 0.04 | 0.14 | 0.05 | 0.23 | 0.29 | 0.57 | 0.78 | 1.48 | 2.60 | 3.48 | 4.90 | 6.07 | 7.69 | 9.79 |
|  |  | Female | 0.07 | 0.02 | 0.03 | 0.01 | 0.04 | 0.10 | 0.09 | 0.12 | 0.27 | 0.32 | 0.45 | 0.64 | 1.12 | 2.02 | 3.01 | 3.32 | 6.10 | 7.89 | 11.17 |
| Breast | 174-175 | Male | 0 | 0 | 0 | 0 | 0 | 0 | 0 | 0.01 | 0 | 0.02 | 0 | 0.05 | 0.02 | 0.08 | 0.09 | 0.18 | 0.15 | 0.11 | 0 |
|  |  | Female | 0 | 0 | 0 | 0.01 | 0.07 | 0.16 | 0.85 | 2.16 | 5.10 | 8.08 | 12.03 | 11.02 | 11.74 | 9.55 | 7.20 | 6.31 | 5.53 | 5.06 | 6.19 |
| Cervix | 180 | Female | 0 | 0 | 0 | 0 | 0.01 | 0.32 | 1.47 | 4.66 | 7.75 | 10.10 | 10.98 | 9.96 | 13.34 | 14.35 | 17.72 | 17.85 | 15.10 | 13.01 | 8.46 |
| Uterus | 179-182 | Female | 0 | 0.03 | 0.03 | 0.08 | 0.24 | 0.26 | 0.47 | 0.59 | 0.65 | 1.42 | 2.24 | 2.32 | 5.00 | 8.96 | 17.56 | 24.74 | 32.98 | 39.22 | 37.62 |
| Ovary | 183 | Female | 0 | 0.03 | 0.03 | 0.08 | 0.24 | 0.25 | 0.43 | 0.48 | 0.51 | 1.05 | 1.30 | 1.25 | 1.50 | 1.34 | 1.78 | 1.56 | 1.86 | 2.56 | 1.28 |
| Prostate | 185 | Female | 0 | 0 | 0 | 0 | 0 | 0 | 0 | 0 | 0.01 | 0.02 | 0.04 | 0.18 | 1.50 | 4.16 | 9.90 | 15.46 | 21.99 | 26.02 | 24.16 |
| Bladder | 188 | Male | 0 | 0 | 0 | 0 | 0 | 0.02 | 0.03 | 0.11 | 0.13 | 0.35 | 0.90 | 0.89 | 2.00 | 3.46 | 5.88 | 7.71 | 9.13 | 10.64 | 12.18 |
|  |  | Female | 0 | 0 | 0 | 0 | 0.02 | 0 | 0 | 0.01 | 0.08 | 0.18 | 0.27 | 0.39 | 1.09 | 1.92 | 2.06 | 3.20 | 4.59 | 4.26 | 3.99 |
| Kidney | 189 | Male | 0.26 | 0.13 | 0 | 0.01 | 0.02 | 0.01 | 0.02 | 0.11 | 0.13 | 0.25 | 0.77 | 0.87 | 1.50 | 2.12 | 3.75 | 3.67 | 4.42 | 4.21 | 3.59 |
|  |  | Female | 0.07 | 0.09 | 0.03 | 0.04 | 0.03 | 0.01 | 0.02 | 0.05 | 0.10 | 0.33 | 0.44 | 0.64 | 1.43 | 2.17 | 3.56 | 4.22 | 4.88 | 6.40 | 4.15 |
| CNS | 191-192 | Male | 0.20 | 0.16 | 0.24 | 0.24 | 0.12 | 0.11 | 0.19 | 0.24 | 0.30 | 0.33 | 0.33 | 0.20 | 0.64 | 0.94 | 0.75 | 1.38 | 1.02 | 1.47 | 1.00 |
|  |  | Female | 0.29 | 0.24 | 0.17 | 0.17 | 0.16 | 0.13 | 0.15 | 0.19 | 0.21 | 0.14 | 0.20 | 0.37 | 0.46 | 0.66 | 0.71 | 0.94 | 0.52 | 0.53 | 0.80 |
| Thyroid | 193 | Male | 0 | 0 | 0 | 0 | 0.05 | 0.13 | 0.22 | 0.27 | 0.23 | 0.38 | 0.45 | 0.45 | 0.67 | 0.38 | 0.30 | 0.40 | 1.26 | 0.42 | 0 |
|  |  | Female | 0.07 | 0 | 0 | 0.07 | 0.16 | 0.48 | 0.93 | 1.12 | 1.33 | 1.24 | 1.20 | 1.58 | 0.99 | 1.21 | 1.41 | 1.21 | 1.22 | 0.96 | 0.48 |
| Hematologic | 200-208 | Male | 0.26 | 1.54 | 0.74 | 0.67 | 1.24 | 0.88 | 0.75 | 0.98 | 1.09 | 1.61 | 2.17 | 2.72 | 4.09 | 5.42 | 8.22 | 10.16 | 13.01 | 12.43 | 15.18 |
|  |  | Female | 0.57 | 1.19 | 0.75 | 0.42 | 0.86 | 0.63 | 0.73 | 0.74 | 1.19 | 1.43 | 1.55 | 2.31 | 3.10 | 4.09 | 5.65 | 6.33 | 7.43 | 7.04 | 4.79 |
| Per 10,000 person-year. | | | | | | | | | | | | | | | | | | | | | |
